# Health Literacy and Health-Related Quality of Life in Beijing Adolescents: A Path Analysis

**DOI:** 10.3928/24748307-20221113-02

**Published:** 2022-10

**Authors:** Shuaijun Guo, Xiaoming Yu, Lucio Naccarella, Rebecca Armstrong, Elise Davis

## Abstract

**Background:**

Health literacy is a critical driver of achieving an equitable world for every child and adolescent. Although the relationship between health literacy and health-related quality of life (HRQoL) has been documented, little is known among adolescents. In addition, due to lack of theory-driven empirical research, it remains unknown about the full relationship between health literacy, its antecedents, and HRQoL.

**Objective:**

This study aimed to apply Manganello's framework to investigate how health literacy was associated with its antecedents and HRQoL in Beijing secondary students.

**Methods:**

A cross-sectional study was conducted with 650 students in years 7 to 9 from four secondary schools in Beijing. Based on Manganello's health literacy framework, a self-administered questionnaire was used to collect information on health literacy, its antecedents (i.e., sociodemographics, self-efficacy, social support, school and community environment), and HRQoL. The 8-item Health Literacy Assessment Tool was used to measure health literacy (score range 0–37), and the KIDSCREEN-10 was used to measure HRQoL (score range 10–50). Path analysis was conducted to examine the mediating role of health literacy in the relationship between its antecedents and HRQoL.

**Key Results:**

Overall, the average score of students' health literacy and HRQoL was 26.37 (±5.89) and 37.49 (±5.78), respectively. Health literacy was positively correlated with HRQoL (*r* = 0.36, *p* < .01). In the final path model, health literacy was not associated with HRQoL. However, students' social support, school environment, and community environment were associated with HRQoL. Health literacy was affected by self-efficacy, social support, and school environment (all *p* < .05).

**Conclusions:**

A range of intrapersonal, interpersonal, and environmental factors were associated with health literacy and HRQoL. A holistic approach is needed to improve health literacy and HRQoL through multilevel intervention strategies such as increasing personal self-efficacy, promoting social support, and creating positive environments. [***HLRP: Health Literacy Research and Practice*. 2022;6(4):e300–e309.**]

**Plain Language Summary:**

We investigated how health literacy was related to its influencing factors and HRQoL among Beijing secondary students in years 7 to 9. Health literacy and HRQoL were independent outcomes affected by a range of social-ecological factors including self-efficacy, social support, and perceptions of school and community environments.

Health literacy represents an individual's ability to find, understand, and use health information to promote and maintain good health (Bröder et al., 2018). It is a personal asset that enables an individual to take control of health determinants and a measurable outcome for national health (Nutbeam, 2000). Mounting evidence shows that low health literacy is associated with a range of adverse health outcomes (Berkman et al., 2011), including frequent use of emergency care, prolonged hospital stays, and high mortality rates, which in turn lead to health disparities (Schillinger, 2020). The World Health Organization recognizes health literacy as a critical driver of achieving an equitable world (Pleasant et al., 2020).

Health-related quality of life (HRQoL) refers to how individuals subjectively assess their own well-being within several dimensions of life, including physical, psychological, and social functions (Riiser et al., 2020). HRQoL is viewed as a comprehensive health indicator of unmet health needs, intervention outcomes, and population health surveys (Zheng et al., 2018). Improving HRQoL across the life course is a central public health goal for across countries (Phyo et al., 2020). As shown in Nutbeam's health promotion outcome model (Nutbeam, 2000), HRQoL can be improved through a variety of health promotion actions, including health literacy interventions that focus on three domains: functional (basic skills in reading and writing health information), interactive (communicating skills to protect health), and critical (appraising health information and applying it into practice).

Although the relationship between health literacy and HRQoL has been documented (Zheng et al., 2018), most studies focus on adults. Adolescents are a relatively healthy population but facing unprecedented health challenges nowadays, with high burdens of disease arising from mental disorders, injury, and violence (Patton et al., 2016). Adolescence is also a critical developmental process of preparing and transitioning to adulthood. During this period, adolescents develop their own self-identity and become more independent about everyday health-related decisions (Kim & White, 2018). As a personal asset, health literacy empowers an individual to make the right health-related actions, resulting in better health outcomes (Guo, Yu, & Okan, 2020; Riiser et al., 2020). Measuring and monitoring health literacy at an early age is crucial to generate long-term better health outcomes from a life course perspective (Guo, Naccarella, et al., 2021).

National and international studies have shown that low health literacy is prevalent among adolescents, ranging from 34% in the United States to 93.7% in China (Guo, 2018; Sansom-Daly et al., 2016). Inadequate health literacy skills may preclude adolescents from adopting health-promoting behaviors and following medical instructions, which in turn lead to poor HRQoL (Ran et al., 2018; Reid et al., 2021; Riiser et al., 2020). While few studies have explored the relationship between health literacy and quality of life (QoL) among adolescents (Ran et al., 2018; Reid et al., 2021; Riiser et al., 2020; Rocha et al., 2017), they mainly focus on the functional domain of health literacy and a broader concept of QoL rather than HRQoL. Also, most existing studies measure health literacy without a conceptual framework, which makes conclusions inconsistent regarding the relationships between health literacy, its antecedents, and HRQoL. A theoretical model is vital to understand what health literacy is and clarify how health literacy relates to other constructs (e.g., antecedents, outcomes) in a holistic way (McCormack et al., 2013).

In the field of adolescent health literacy, more than 20 theoretical models have been proposed (Bröder et al., 2017). In the present study, we used Manganello's framework (Manganello, 2008) as a guide to understand how adolescent health literacy was related to its antecedents and HRQoL in Chinese students (See Appendix 1 for detailed rationales: https://osf.io/tew9f/). Manganello's framework has three main modules: (1) antecedents (i.e., factors at intrapersonal, interpersonal, and environmental levels) that may influence health literacy; (2) the construct of health literacy, which consists of functional, interactive and critical domains; and (3) health-related outcomes (e.g., HRQoL) (Manganello, 2008). This framework was chosen because of two reasons. First, it considers the unique characteristics of adolescents, who are mainly dependent on their parents, friends and peers for making health-related decisions (Bröder et al., 2020). Second, this framework is well-supported by empirical evidence on the relationships between health literacy, its antecedents and HRQoL. Previous studies indicated that both health literacy and HRQoL were influenced by either intrapersonal factors such as family socioeconomic status (Demir & Leyendecker, 2018; Mao et al., 2020) and personal self-efficacy (Goldstein-Leever et al., 2020; Guo, Naccarella, et al., 2020), or interpersonal factors such as social support (Demir & Leyendecker, 2018; Guo, Naccarella, et al., 2021) or environmental factors such as school environment (Gaspar et al., 2012; Smith et al., 2021) and community environment (Guo, Yu, Davis, et al., 2020; Yeung & Towers, 2014). Health literacy is also suggested as a mediating variable between socioeconomic status and HRQoL in a recent literature review (Stormacq et al., 2019).

In the present study, we aimed to apply Manganello's framework to investigate the pathway linking a range of antecedents through health literacy to HRQoL among Beijing adolescents. We proposed the following three hypotheses: (1) Hypothesis 1: Health literacy would be an outcome influenced by factors at intrapersonal, interpersonal and environmental levels; (2) Hypothesis 2: HRQoL would be an outcome influenced by factors at intrapersonal, interpersonal and environmental levels; and (3) Hypothesis 3: Health literacy would be a mediating variable between its antecedents (e.g., intrapersonal, interpersonal and environmental factors) and HRQoL. We focused on school settings rather than health care contexts because school is the primary place to develop and improve adolescent health literacy (Nash et al., 2021).

## Methods

### Participants and Procedure

The current study is part of a PhD research project (Guo, 2018). A cross-sectional study was designed to recruit adolescents from secondary schools in two districts of Beijing, China, using convenience sampling. Two districts were selected according to their socioeconomic levels, one representing high and the other representing low. Two schools in each district were selected based on previous research partnerships and appropriate survey timing (class time, class break time or lunchtime). At each school, students in two whole classes (ranging from 20 to 35 students) from each year level (years 7, 8 and 9) were invited to complete a self-administered questionnaire. Passive, opt-out consent was obtained from both parents and students. In total, 661 students were invited to participated in the study. As suggested by Raykov and Marcoulides (2012), a minimum sample size for path analysis should be at least 10 times of the number of free parameters of the model. In our case here, the number of estimated parameters was 48. As such, our sample size was larger than the recommended minimum sample size of 480. Data collection was undertaken in November 2015 and was approved by the ethics committee of Peking University (Ethics ID: IRB00001052-15024) and The University of Melbourne (Ethics ID: 1442884).

### Questionnaire

**Figure 1.**
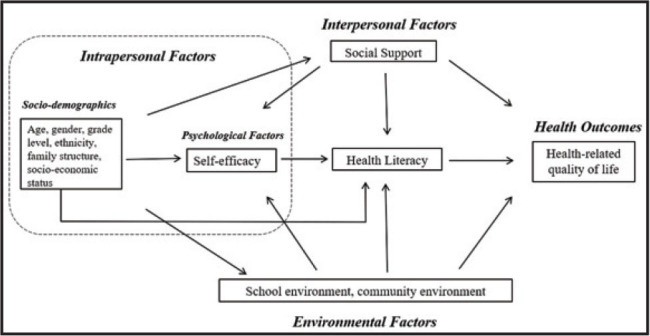
The hypothesized model depicting the relationship between adolescent health literacy and health-related quality of life.

According to **Figure 1**, we designed a questionnaire to measure students' health literacy, key antecedents and HRQoL (See Appendix 2 for detailed measures: https://osf.io/tew9f/).

### Intrapersonal Factors

Intrapersonal factors included sociodemographics and self-efficacy. Sociodemographics included age, gender (male or female), ethnicity (Han or ethnic minorities [Hui, Chaoxian, Menggu]), year level (years 7, 8 or 9), family structure (living with two biological parents or other living arrangements), and family affluence level measured by the Family Affluence Scale (low, medium or high) (Liu et al., 2012). Personal self-efficacy was measured using the General Self-Efficacy Scale (GSES) (Schwarzer & Jerusalem, 1995), a 10-item scale that assesses personal belief in the ability to cope with a variety of life challenges. Respondents indicated their level of agreement on a 4-point scale (1 = not at all true, 4 = exactly true). The GSES is available in Chinese and has strong structural validity and excellent internal consistency (Schwarzer et al., 1997). The GSES total score range is 10 to 40, with higher scores indicating higher levels of self-efficacy.

### Interpersonal Factors

Interpersonal factors were assessed using the Multidimensional Scale of Perceived Social Support (MSPSS) (Zimet et al., 1988), a 12-item scale that measures an individual's perceived support from family, friends and significant others. Respondents answered each item on a 7-point Likert scale (1 = *very strongly disagree*, 7 = *very strongly agree*). The MSPSS has been validated in Chinese adolescents, showing high internal consistency, satisfactory concurrent validity, and construct validity (Chou, 2000). The MSPSS total score range is 12 to 84, with higher scores reflecting higher levels of social support.

### Environmental Factors

School environment was assessed using the School Environment Scale (SES), which is derived from the Communities That Care Youth Survey (Glaser et al., 2005). The SES consists of ten items measuring students' subjective feelings about opportunities and rewards for prosocial involvement at school. Respondents indicated their level of agreement with each statement on a 4-point Likert scale (1 = *strongly disagree*, 4 = *strongly agree*). In the present study, the SES showed high internal consistency (Cronbach's alpha = 0.88) and satisfactory construct validity (comparative fit index [CFI] = 0.095∼0.996, root mean error of approximation [RMSEA] = 0.048∼0.053). The SES total score range is 10 to 40, with higher scores suggesting stronger bonds of attachment to school.

Community environment was assessed using the Community Environment Scale (CES), which measures respondents' subjective feelings of their neighborhood environment such as cleanliness and safety (Gray & Sanson, 2005). The CES consists of nine items in three domains: neighborhood livability, neighborhood facilities, and traffic. Participants answered each item on a 5-point scale (1 = *strongly disagree*, 4 = *strongly agree*; 0 = *do not know*). The CES showed adequate internal consistency (Cronbach's alpha = 0.84) and satisfactory construct validity (factor analysis indicated a three-factor construct and explained 67.78% of the total variance) in the present study. The CES total score range is 0 to 36, with higher scores indicating a more livable and supportive community.

### Health Literacy

Health literacy was assessed using the Chinese version 8-item Health Literacy Assessment Tool (HLAT-8) that measures an individual's ability to access, understand, evaluate, and communicate health information in everyday life (Guo et al., 2018). The HLAT-8 total score range is 0 to 37, with higher scores indicating higher levels of health literacy. The HLAT-8 has been validated in Chinese secondary students, showing satisfactory reliability and strong validity (Guo et al., 2018).

### HRQoL

HRQoL was measured by the KIDSCREEN-10 developed by Ravens-Sieberer et al. (2010). The KIDSCREEN-10 is a short version of the KIDSCREEN-52 that assesses the health-related quality of life of healthy children and children and adolescents who are chronically ill and adolescents age 8 to 18 years. Respondents answered each item on a 5-point Likert scale (1 = *not at all/never*, 5 = *extremely/always*). The KIDSCREEN-10 has high internal consistency (Cronbach's alpha = 0.79) and strong structural validity (χ^2^/*df* = 2.877, C*FI* = 0.959, RMS*EA* = 0.055) in our sample. The KIDSCREEN-10 score is obtained by reversing the scores on two items (1 = 5, 2 = 4, and so on) and then summing scores across all ten items. The total score ranges from 10 to 50, with higher scores indicating higher levels of HRQoL.

### Data Analysis

All statistical analyses were conducted using SPSS 22.0 and AMOS 23.0. Descriptive statistics (frequency/percentage, mean, median) were used to examine participants' sociodemographic and each scale. Univariate analysis (*t*-test, ANOVA, nonparametric test) and correlation analysis (Pearson and Spearman correlation analysis) were conducted before path analysis which was performed using the maximum likelihood method. Model fit was examined with the relative chi-square goodness-of-fit statistic (χ^2^/*df*), CFI), Tucker and Lewis's Index of Fit (TLI) and RMSEA. An acceptable model fit was considered when the χ^2^/*df* statistic ≤3, CFI values ≥0.95, TLI values ≥0.95 and RMSEA values ≤0.08 (Kline, 2016).

The individual mean substitution was conducted for non-response items in self-report scales. The percentages of missing items for the GSES, MSPSS, SES, CES, HLAT-8, and KIDSCREEN-10 ranged from 0.9% to 1.8%, 0.9% to 2.0%, 0.9% to 1.7%, 2.5% to 2.9%, 0.2% to 0.6%, and 0% to 0.3%, respectively. Data normality was assessed using skewness and kurtosis values.

## Results

### Sample Characteristics and Descriptive Statistics of Each Scale

In total, 661 students were invited to participate, with 11 students declining—a response rate of 98.3% (650/661). The mean age of participants was 13.42 ± 1.01 (range: 11–17 years). The distributions of gender, year level, ethnicity, family structure, family affluence level, and descriptive statistics of each scale are shown in **Table 1**. Overall, the average score of students' health literacy and HRQoL was 26.37 (±5.89) and 37.49 (±5.78), respectively.

**Table 1:**
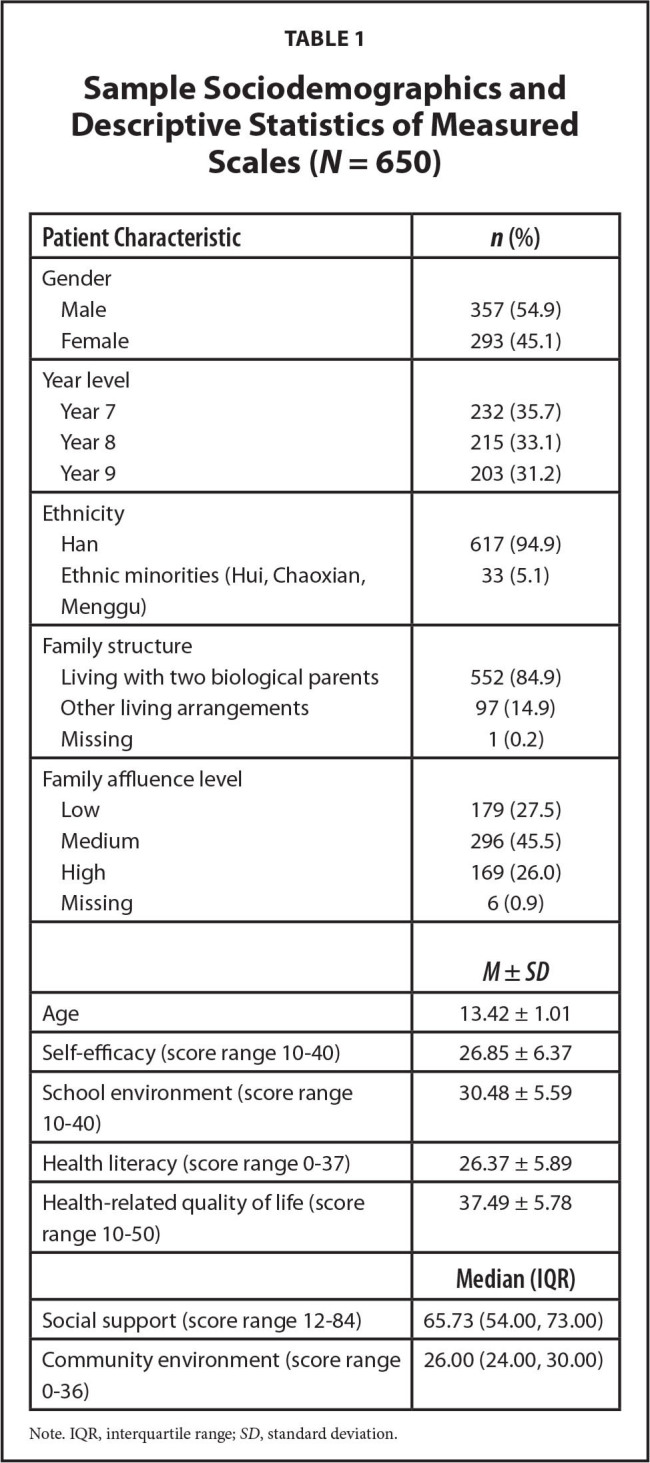
Sample Sociodemographics and Descriptive Statistics of Measured Scales (*N* = 650)

**Patient Characteristic**	***n* (%)**

Gender	
Male	357 (54.9)
Female	293 (45.1)

Year level	
Year 7	232 (35.7)
Year 8	215 (33.1)
Year 9	203 (31.2)

Ethnicity	
Han	617 (94.9)
Ethnic minorities (Hui, Chaoxian, Menggu)	33 (5.1)

Family structure	
Living with two biological parents	552 (84.9)
Other living arrangements	97 (14.9)
Missing	1 (0.2)

Family affluence level	
Low	179 (27.5)
Medium	296 (45.5)
High	169 (26.0)
Missing	6 (0.9)

	***M* ± *SD***

Age	13.42 ± 1.01

Self-efficacy (score range 10–40)	26.85 ± 6.37

School environment (score range 10–40)	30.48 ± 5.59

Health literacy (score range 0–37)	26.37 ± 5.89

Health-related quality of life (score range 10–50)	37.49 ± 5.78

	**Median (IQR)**

Social support (score range 12–84)	65.73 (54.00, 73.00)

Community environment (score range 0–36)	26.00 (24.00, 30.00)

Note. IQR, interquartile range; *SD*, standard deviation.

### Relationships Between Health Literacy, its Antecedents, and HRQoL

**Table 2** shows that there are differences in scores of self-efficacy, social support, school environment, community environment and health literacy by gender, year level, family structure and family affluence level. Correlation analysis showed that students' health literacy was positively correlated with self-efficacy, social support, school environment, community environment and HRQoL (*r* = 0.25–0.61, *p* < .01) (See **Table 3**).

**Table 2:**
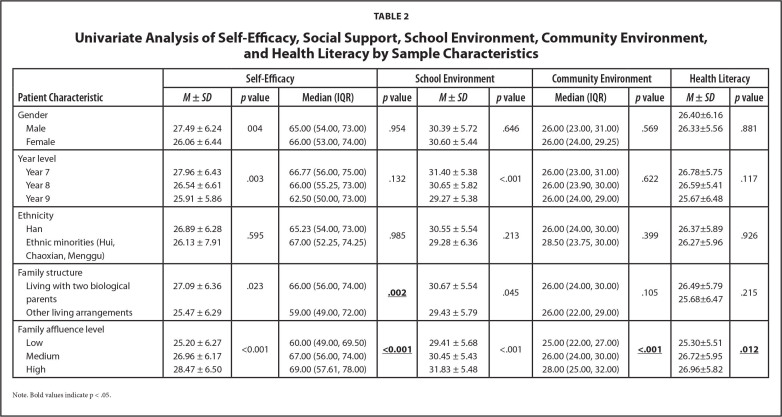
Univariate Analysis of Self-Efficacy, Social Support, School Environment, Community Environment, and Health Literacy by Sample Characteristics

**Patient Characteristic**	**Self-Efficacy**			**School Environment**			**Community Environment**		**Health Literacy**	
			
	***M* ± *SD***	***p* value**	**Median (IQR)**	***p* value**	***M* ± *SD***	***p* value**	**Median (IQR)**	***p* value**	***M* ± *SD***	***p* value**

Gender										
Male	27.49 ± 6.24	004	65.00 (54.00, 73.00)	.954	30.39 ± 5.72	.646	26.00 (23.00, 31.00)	.569	26.40±6.16	.881
Female	26.06 ± 6.44		66.00 (53.00, 74.00)		30.60 ± 5.44		26.00 (24.00, 29.25)		26.33±5.56	

Year level										
Year 7	27.96 ± 6.43	.003	66.77 (56.00, 75.00)	.132	31.40 ± 5.38	<.001	26.00 (23.00, 31.00)	.622	26.78±5.75	.117
Year 8	26.54 ± 6.61		66.00 (55.25, 73.00)		30.65 ± 5.82		26.00 (23.90, 30.00)		26.59±5.41	
Year 9	25.91 ± 5.86		62.50 (50.00, 73.00)		29.27 ± 5.38		26.00 (24.00, 29.00)		25.67±6.48	

Ethnicity										
Han	26.89 ± 6.28	.595	65.23 (54.00, 73.00)	.985	30.55 ± 5.54	.213	26.00 (24.00, 30.00)	.399	26.37±5.89	.926
Ethnic minorities (Hui, Chaoxian, Menggu)	26.13 ± 7.91		67.00 (52.25, 74.25)		29.28 ± 6.36		28.50 (23.75, 30.00)		26.27±5.96	

Family structure										
Living with two biological parents	27.09 ± 6.36	.023	66.00 (56.00, 74.00)	**.002**	30.67 ± 5.54	.045	26.00 (24.00, 30.00)	.105	26.49±5.79	.215
Other living arrangements	25.47 ± 6.29		59.00 (49.00, 72.00)		29.43 ± 5.79		26.00 (22.00, 29.00)		25.68±6.47	
** **										
Family affluence level										
Low	25.20 ± 6.27	<0.001	60.00 (49.00, 69.50)	**<0.001**	29.41 ± 5.68	<.001	25.00 (22.00, 27.00)	**<.001**	25.30±5.51	**.012**
Medium	26.96 ± 6.17		67.00 (56.00, 74.00)		30.45 ± 5.43		26.00 (24.00, 30.00)		26.72±5.95	
High	28.47 ± 6.50		69.00 (57.61, 78.00)		31.83 ± 5.48		28.00 (25.00, 32.00)		26.96±5.82	

Note. Bold values indicate p < .05.

**Table 3:**
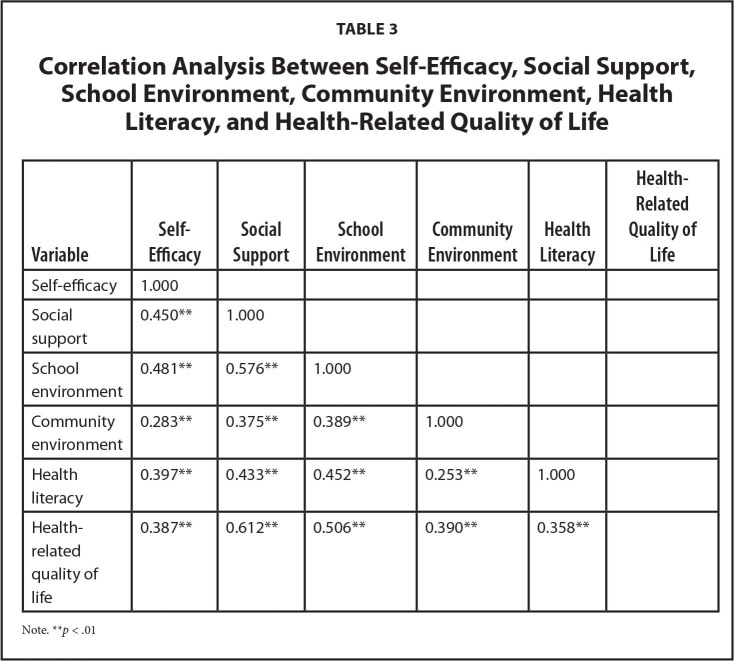
Correlation Analysis Between Self-Efficacy, Social Support, School Environment, Community Environment, Health Literacy, and Health-Related Quality of Life

**Variable**	**Self-Efficacy**	**Social Support**	**School Environment**	**Community Environment**	**Health Literacy**	**Health-Related Quality of Life**
Self-efficacy	1.000					
Social support	0.450^**^	1.000				
School environment	0.481^**^	0.576^**^	1.000			
Community environment	0.283^**^	0.375^**^	0.389^**^	1.000		
Health literacy	0.397^**^	0.433^**^	0.452^**^	0.253^**^	1.000	
Health-related quality of life	0.387^**^	0.612^**^	0.506^**^	0.390^**^	0.358^**^	

Note.

** *p* < .01

### Path Analysis Examining the Mediating Role of Health Literacy

**Figure 2.**
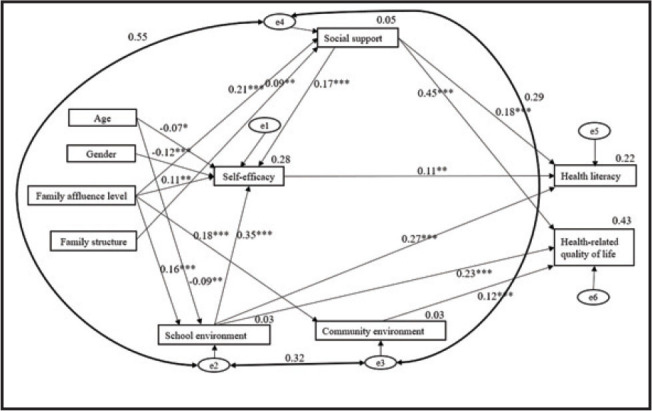
The final path model linking adolescent health literacy and health-related quality of life. Coefficients are standardized path coefficients. The error term (e1–e6) is the residual term, representing the margin of error within a statistical model and providing an explanation for the difference between the results of the model and actual observed results.

After univariate and correlation analyses, all significant independent variables related to health literacy or HRQoL were considered for next-step path analysis. The original HRQoL path model demonstrated poor data fit: χ^2^/*df* (23, *N* = 625) = 14.650, *p* < .001, C*FI* = 0.711, T*LI* = 0.434, RMS*EA* = 0.148 (90% confidence interval [CI]: 0.134–0.162), and the path from health literacy to HRQoL was non-significant (Beta = 0.004, *p* = .905). Although the path from health literacy to HRQoL was not significant in the original model, there were significant relationships between other variables. Examination of modification indices suggested that the model fit could be improved by connecting errors between social support and school environment, between school environment and community environment, and between social support and community environment (See Appendix 3: https://osf.io/tew9f/), represented by the bold, double-headed arrows in the trimmed model in **Figure 2**. These modifications were made based on the social ecological theory (Higgins et al., 2009), which suggests that social support, school environment, and community environment interact with each other and contribute to students' health literacy. The final trimmed HRQoL path model showed satisfactory data fit: χ^2^/df (26, *N* = 625) = 1.624, *p* = .023, C*FI* = 0.985, T*LI* = 0.974, RMS*EA* = 0.032 (90% CI: 0.012–0.048).

In the final trimmed HRQoL path model, there were significant and direct paths from social support (Beta = 0.45, *p* < .001), school environment (Beta = 0.23, *p* < .001) and community environment (Beta = 0.12, *p* < .001) to HRQoL. Similarly, there were significant and direct paths from self-efficacy (Beta = 0.11, *p* = .007), social support (Beta = 0.18, *p* < .001) and school environment (Beta = 0.27, *p* < .001) to health literacy. Additional significant paths are presented in Appendix 4: https://osf.io/tew9f/. Based on the squared multiple correlation coefficients (R2), the final trimmed model explained 22% of the variance in health literacy and 43% of the variance in HRQoL.

## Discussion

We used cross-sectional data to investigate the relationship between health literacy, its antecedents and HRQoL among Beijing secondary students based on Manganello's health literacy framework. Specifically, there were three key findings. First, a range of intrapersonal, interpersonal, and environmental factors were associated with health literacy. Second, intrapersonal, interpersonal, and environmental factors have direct and indirect relationships with HRQoL. Third, health literacy was related to HRQoL in correlation analysis, but not found as a mediating variable between its antecedents and HRQoL.

Corresponding to our Hypothesis 1, we found that students' health literacy was affected by self-efficacy, social support, and school environment. Our finding is consistent with other health literacy theoretical frameworks such as the social-ecological model (Higgins et al., 2009) and the health promoting school model (Deal & Hodges, 2009), suggesting that low health literacy is not only an individual's issue, but results from interactions with the broader environment. We found that the effects of school environment and social support are greater than the effect of self-efficacy. Adolescents are experiencing a life stage in which cognitive and social development processes take place (Bröder et al., 2020). Therefore, they are more likely to seek support from peers and parents when addressing health issues (Bröder et al., 2020). In addition, adolescents spend most of their daytime in schools where they learn health knowledge and health literacy skills (Nash et al., 2021). Intervening on intrapersonal factors such as personal self-efficacy, family structure and family affluence is also important, because they serve as foundations for the social determinants of health. To improve adolescent health literacy, a whole school approach is needed for the school-based intervention program that integrates strategies such as enhancing personal self-efficacy, improving social support, and creating supportive school environments.

Aligning with previous studies (Demir & Leyendecker, 2018; Gaspar et al., 2012) and our Hypothesis 2, we found students' HRQoL was determined by social support, school environment and community environment. Particularly, social support is a stronger influencing factor of HRQoL than school and community environments. This highlights the importance of healthy psychosocial development to adolescents' HRQoL. The structure and functions of social support are closely related to personal adjustment and social skills against stressful life events, thus reducing the tension and increasing life satisfaction (Gaspar et al., 2012). In high-affluence families, children are more connected with social networks (Bi et al., 2021), community resources and facilities (Lenzi et al., 2012), as well as educational opportunities at school (Li & Qiu, 2018), which in turn contribute to higher levels of life satisfaction (Chang et al., 2020; Horanicova et al., 2022; Tapia-Fonllem et al., 2020). The environment where students are living is an independent factor that affects one's HRQoL. As shown in a recent study investigating the relationship between environmental factors and HRQoL, Chang et al. (2020) found that several pathways (e.g., stress and sleep) may explain the relationship between environmental factors to HRQoL among community residents. However, it is out of scope in the present study. Future research may consider exploring these complex pathways from environment factors to HRQoL and informing interventions for school-aged adolescents. In response to low levels of HRQoL amongst students, our findings suggest a need for a multilevel intervention strategy that involves the collaboration and engagement with families, schools and communities.

In response to our Hypothesis 3, we did not observe the mediating role of health literacy in the relationship between its antecedents and HRQoL. Our finding is not consistent with those from other similar studies when using health behaviours or health status as the outcome of interest (Guo, Naccarella, et al., 2021; Guo, Yu, et al., 2021). There are several reasons that may explain this difference. First, HRQoL is a distinct construct from health behaviours and health status. As shown in Nutbeam's health promotion outcome model (Nutbeam, 2000), HRQoL is a distal health outcome resulting from health behaviours. While HRQoL and health status are often used interchangeably in the literature, HRQoL is more related to one's mental health than physical functioning, whereas health status is more related to one's physical functioning (Smith et al., 1999). Second, other unmeasured factors such as patient-provider relationship and self-care may explain the pathway from health literacy to HRQoL (Lee et al., 2016; Paasche-Orlow & Wolf, 2007). Third, previous studies showed that the relationship between health literacy and HRQoL was stronger among older age groups than younger age groups (Zheng et al., 2018). However, in the correlation analysis, we found a positive relationship between health literacy and HRQoL in our sample (*r* = 0.36, *p* < .01). This finding is consistent with a recent meta-analysis showing the relationship between health literacy and HRQoL among healthy populations (*r* = 0.35, *p* < .01) (Zheng et al., 2018).

Although we used Manganello's health literacy framework as a guide to enhance the rigor, transparency and clarity of the current research, there are several limitations. First, this study only used cross-sectional data to examine the pathways from key antecedents through health literacy to HRQoL at a single time point. Longitudinal cohort studies are needed in future to replicate our findings. Second, convenience sampling may limit the generalizability of our findings. We recruited students from four secondary schools in a metropolitan city where the ability of subjects to access good education might be much higher than the general population. Future studies are recommended to recruit adolescents from a wider range of socio-demographic backgrounds. Third, this study did not consider students' disease characteristics, function status and global health perceptions, which are essential pathway factors of HRQoL (Duangchan & Matthews, 2021). Fourth, the standardized correlation coefficients in the final trimmed path model were small (ranging from 0.03 to 0.45), suggesting weak to moderate relationships between variables. While path analysis is useful to examine theory-driven hypotheses, it is often based on several assumptions (e.g., no measurement error, linearity) (Garson, 2013). Therefore, findings should be interpreted within the context and these assumptions. Finally, self-report bias may exist for health literacy measurement among our target population who are healthy adolescents. Future research may consider using different formats of data collection (e.g., objective measures such as the Newest Vital Sign [Linnebur & Linnebur, 2018]) to verify whether such measurement bias exists. Similarly, self-report bias may exist for other measurement scales including HRQoL. However, we used well-established and valid instruments in the present study to minimize the extent of such bias.

## Conclusion

We found that Manganello's health literacy framework was partly supported by empirical data. Adolescent health literacy and HRQoL are independent outcomes influenced by a set of social-ecological factors: self-efficacy, social support, and perceptions of school and community environments. Our findings suggest that a holistic approach is needed to improve health literacy and HRQoL through multilevel intervention strategies such as increasing personal self-efficacy, promoting social support, and creating positive environments.
